# Hängen die Stundenkontingente für ambulante Psychotherapie zur Behandlung von Depressionen mit deren Schweregrad zusammen?

**DOI:** 10.1007/s00115-022-01374-3

**Published:** 2022-08-10

**Authors:** Susanne Singer, Julian Blanck, Ida Scholz, Matthias Büttner, Lena Maier

**Affiliations:** 1 Abt. Epidemiologie und Versorgungsforschung, Institut für Medizinische Biometrie, Epidemiologie und Informatik (IMBEI), Universitätsmedizin der Johannes Gutenberg-Universität Mainz, Obere Zahlbacher Straße 69, 55131 Mainz, Deutschland; 2 Mainz, Deutschland

**Keywords:** Schwere der Erkrankung, Dosis, Affektive Erkrankungen, Versorgungsforschung, Politik, Severity, Dose, Affective Disorders, Healthcare research, Health policy

## Abstract

**Fragestellung:**

Vor dem Hintergrund der Debatte um eine mögliche Festlegung des Leistungsumfangs für ambulante Psychotherapie anhand der Diagnose haben wir untersucht, ob in der aktuellen Versorgungspraxis bei Patienten mit Depressionen die Anzahl der beantragten sowie die der vom Gutachter befürworteten Stunden mit dem Schweregrad der Erkrankung zusammenhängen.

**Methode:**

Aus einer Zufallsstichprobe von 1000 Anträgen auf Kostenübernahme für ambulante analytische oder tiefenpsychologisch fundierte Psychotherapie wurden jene herausgesucht, in denen eine Depressionsdiagnose mit Schweregradeinschätzung (ICD-10 F32 oder F33) entweder im Bericht oder auf dem Antragsformular kodiert worden war. Die Zahl der beantragten und der befürworteten Stunden pro Antrag wurde extrahiert. Bei Umwandlungs- und Fortführungsanträgen wurden dabei auch die im Vorfeld stattgefundenen Therapiestunden berücksichtigt. Ein möglicher Zusammenhang des Schweregrads der Depression mit der Zahl der beantragten bzw. der vom Gutachter befürworteten Stunden wurde anhand von Spearman-Rangkorrelationen überprüft.

**Ergebnisse:**

Insgesamt 521 Anträge (52 %) enthielten eine F32- und/oder eine F33-Diagnose. Davon waren 63 (12 %) als leicht kodiert, 349 (67 %) als mittelgradig und 50 (10 %) als schwer.

Im Median wurden 75 h bei leichter sowie je 50 h bei mittelgradiger bzw. bei schwerer Depression beantragt, wobei die Zahlen innerhalb der Gruppen stark variierten (10 bis 327 h) und der Zusammenhang zwischen Schweregrad und beantragter Stundenzahl gering war (Rho −0,10).

Die befürworteten Stundenkontingente waren im Median 74 (leichte Depression), 50 (mittelgradig) und 50 (schwer) Stunden, auch hier war die Spannweite hoch (0 bis 327 h) und die Korrelation gering (Rho −0,11).

**Diskussion:**

Es gibt keine Anhaltspunkte dafür, dass Psychotherapeuten die benötigten Stundenkontingente allein anhand des Schweregrads der Diagnose festlegen.

## Abstract (en)

**Objective:**

This study investigated how the number of sessions for outpatient psychodynamic psychotherapy among patients with depressive disorders is associated with the severity of the disease.

**Methods:**

From a random sample of 1000 applications for reimbursement of outpatient psychodynamic psychotherapy, we selected those in which a diagnosis of depression with a severity rating (ICD-10 F32 or F33) was coded.

The association of levels of depression severity (mild, moderate, and severe) with the number of sessions requested and the number of sessions endorsed by the reviewer was investigated using Spearman’s rank correlation. If the application was for a continuation of an ongoing therapy, the previous sessions were taken into account too.

**Results:**

A total of 521 (52%) applications contained a diagnosis of F32 and/or F33. Out of these, 63 (12%) were coded as mild, 349 (67%) as moderate, and 50 (10%) as severe.

There were 75 sessions applied for in patients with mild depression (median), 50 in patients with moderate depression, and 50 in patients with severe depression, whereby the range within each severity group was considerable (10 to 327 sessions) and the correlation was low (Rho −0.10).

The median number of endorsed sessions was 74 in patients with mild depression, 50 in patients with moderate, and 50 in patients with severe depression. Here, too, the range was high (0 sessions to 327 sessions) and the association weak (Rho −0.11).

**Discussion:**

There is no evidence that psychotherapists determine the necessary number of sessions solely based on the severity of the diagnosis.

Im Jahr 2021 wurde in Deutschland unter dem Stichwort „Rasterpsychotherapie“ diskutiert, ob die Stundenkontingente für ambulante Psychotherapie anhand der Diagnose der psychischen Erkrankung bemessen werden könnten. Einer entsprechenden geplanten Gesetzesänderung wurde von vielen Seiten aufgrund inhaltlicher und klinischer Überlegungen Widerstand entgegengebracht. In der vorliegenden Studie sollte nun empirisch untersucht werden, ob Daten aus der Versorgungspraxis eher für oder eher gegen ein solches Vorgehen sprechen.

## Einleitung

Der Bedarf an ambulanter Psychotherapie ist anhaltend hoch [7, 11, 14, 20], während die finanziellen Ressourcen der kassenärztlichen Versorgung begrenzt sind. Psychotherapeutische Behandlungen stehen dabei in Konkurrenz zu anderen Gesundheitsleistungen. Gegenwärtig müssen sich Patienten daher auf aufwändige Suchprozesse und lange Wartezeiten auf einen Psychotherapieplatz einstellen [4, 5, 18, 20]. Eine aktuelle Studie [21] zeigt, dass bis zu 50 Telefonate geführt werden, um eine Behandlung beginnen zu können, ein Teil der Patienten gibt die Suche auf (7 %) oder beginnt diese gar nicht erst (20 %).

Diese Versorgungssituation fordert politische Entscheidungsträger zu Verbesserungen auf. Eine Idee, die im Jahr 2021 in Deutschland öffentlich diskutiert wurde, war eine Kontingentierung der Psychotherapie anhand der Diagnose der psychischen Erkrankung („bedarfsgerecht und schweregradorientiert“), offenbar in der – nicht unwidersprochenen – Annahme, dadurch Behandlungskapazitäten gewinnen zu können. Dies sollte via Änderungsantrag 49 zum Gesundheitsversorgungs-Weiterentwicklungsgesetz (GVWG) umgesetzt werden, aber Patienten, Psychotherapeuten und Ärzte setzten dem Widerstand entgegen, sodass es vorerst nicht zu der Gesetzesänderung kam.

Unabhängig von politischen Interessen stellt sich jedoch die Frage, ob dieser Vorschlag sachgerecht war. Um dies beurteilen zu können, sollten möglichst empirische Daten genutzt werden, wobei einer der Bezugspunkte die gegenwärtige Versorgungspraxis ist (wohl wissend, dass das Gegenwärtige nicht unbedingt „das Beste“ ist). Deshalb sollte in der vorliegenden Studie untersucht werden, ob in der bisherigen psychotherapeutischen Versorgungspraxis die Anzahl der beantragten und die der befürworteten Therapiestunden mit dem Schweregrad der Erkrankung zusammenhängen. Depressionen wurden dafür als Analyseeinheit gewählt, weil sie die häufigsten Erkrankungen in der ambulanten psychotherapeutischen Versorgung sind [7, 9, 14] und weil bei diesen Diagnosen regulär immer ein Schweregrad kodiert werden muss. Einen Unterschied von 25 h definierten wir vorab als relevant, da dies bis zur Psychotherapiestrukturreform dem Umfang einer Kurzzeittherapie entsprach. Unsere Hypothese war, dass weder die Anzahl der beantragten noch die der vom Gutachter befürworteten Stunden mit dem Schweregrad der Depressionsdiagnose zusammenhängen.

## Methoden

### Studiendesign

Es handelt sich um eine retrospektive Kohortenstudie basierend auf Routinedaten, nämlich auf Anträgen zur Kostenübernahme für Psychotherapie in psychoanalytisch begründeten Verfahren an Krankenkassen in ganz Deutschland.

Das Studienprotokoll wurde zu Beginn der Arbeit der zuständigen Ethikkommission der Landesärztekammer Rheinland-Pfalz vorgelegt, welche keine berufsethischen oder berufsrechtlichen Bedenken erhob (Ethikvotum # 2018-13221).

### Material

Aus einem Pool von ca. 40.000 Anträgen auf Kostenübernahme für tiefenpsychologisch fundierte bzw. analytische Psychotherapie aus den Jahren 2003 bis 2017 zogen wir eine nach Antragsjahr geschichtete Zufallsstichprobe von 1000 Anträgen. Die Anträge liegen als gescannte PDF-Dateien vor und beinhalten sowohl die Formulare entsprechend Psychotherapievereinbarung (PTV) als auch den Bericht für den Gutachter.

Die gesampelten Dateien wurden von Projektmitarbeitern nach Unterzeichnung einer Verpflichtung zur Einhaltung des Datengeheimnisses nach §8 Landesdatenschutzgesetz alle einzeln durchgesehen und mögliche personenidentifizierende Angaben geschwärzt. Dies konnte z. B. ein Stempel mit dem Namen der Therapeutin sein oder die Chiffre des Antrags (denn sie enthält den ersten Buchstaben des Namens). Mitunter hatten die Therapeuten auch den Klarnamen des Patienten auf den Bericht geschrieben, obwohl das nicht vorgesehen ist. Die solcherart geschwärzten Anträge wurden auf einem sicheren Server gespeichert.

### Datenextraktion

Es wurden Angaben aus den PTV-Formularen (unter anderem die ICD-10 F-Diagnosen, die Zahl der beantragten Therapiestunden, die Zahl der befürworteten Stunden, die Zahl der bisherigen Stunden, die Abrechnungsgenehmigung(en) des Therapeuten) und Angaben aus dem Bericht (unter anderem biografische Angaben und soziodemografische Merkmale des Patienten sowie die hier genannten ICD-10-Diagnosen) extrahiert.

### Stichprobe

Für die geplante Analyse wurden alle Anträge ausgewählt, in denen mindestens eine Diagnose mit der Ziffer F32 oder F33 vergeben worden war, denn hier sind regelhaft die Schweregrade (leicht, mittelgradig, schwer, schwer mit psychotischen Symptomen) mit kodiert. Alle Diagnosenennungen wurden berücksichtigt, sei es auf dem PTV-Formular oder im Bericht an den Gutachter. Hierbei konnte nicht zwischen Haupt- und Nebendiagnosen unterschieden werden, weil dies in den Anträgen in der Regel nicht entsprechend gekennzeichnet wurde (die Reihenfolge der Diagnosen konnte dabei auch nicht als Indiz für die „Wichtigkeit“ genommen werden, da diese mitunter zwischen PTV-Bogen und Bericht variierte). Es wurden sowohl Anträge auf Gruppen- als auch auf Einzeltherapie eingeschlossen.

### Berechnung der Stundenkontingente

Bei Erstanträgen verwendeten wir die in diesem ersten Antragsschritt beantragten Stunden für die Auswertungen. Bei Umwandlungsanträgen wurden die beantragten Stunden plus 25 h berechnet, da davon auszugehen ist, dass vorher immer mindestens 25 h beantragt und bewilligt worden waren. Bei Fortführungsanträgen muss auf den PTV-Formularen immer die Anzahl der bisherigen Stunden eingetragen sein, diese wurden zu den aktuell beantragten addiert. Mit diesem Vorgehen kann man davon ausgehen, dass es sich bei den analysierten Stunden jeweils um das **Minimum** der beantragten bzw. befürworteten Kontingente handelt, über das Maximum kann man keine Aussagen machen, da ggf. weitere Umwandlungs- oder Fortführungsanträge nach dem Zeitpunkt des vorliegenden Antrags bei den Kassen eingereicht wurden.

### Statistische Analyse

Eine neue Variable „Schweregrad“ wurde kreiert, die sich aus der Ziffer nach dem Punkt aus der ICD-10-Diagnose ergab (also 0, 1, 2 oder 3; Diagnosen mit den Ziffern 4, 8 oder 9 nach dem Punkt wurden in den Schweregrad-Vergleichen nicht berücksichtigt).

Aufgrund der Bewilligungsschritte war von einer Nicht-Normalverteilung der beantragten und befürworteten Stunden auszugehen, weshalb der Zusammenhang von Schweregrad mit der Stundenzahl anhand von nicht-parametrischen Spearman-Rangkorrelationen untersucht wurde. Die Voraussetzungen zur Durchführung dieses Tests wurde anhand von Histogramm-Plots überprüft. Zusätzlich wurden Equality-of-Median-Tests durchgeführt.

Bei diesen Zusammenhangsanalysen wurden Anträge auf tiefenpsychologisch fundierte Psychotherapie und Anträge auf analytische Psychotherapie primär gemeinsam betrachtet, da davon auszugehen ist, dass Stundenkontingente nicht so sehr eine Folge des gewählten Verfahrens sind, sondern eher anders herum, dass die Therapeuten dem Patienten das Verfahren vorschlagen auch in Abhängigkeit davon, welches Stundenkontingent als notwendig erachtet wird. Die Korrelationen werden darüber hinaus zur Information auch getrennt für die beiden Verfahren dargestellt.

In einer Sensitivitätsanalyse führten wir die gleichen Analysen nur mit den Erstanträgen durch, um zu prüfen, ob sich die Ergebnisse von der kombinierten Auswertung (Erst‑, Umwandlungs- und Fortführungsanträge) unterscheiden. Eine weitere Sensitivitätsanalyse schloss alle Patienten aus, bei denen eine komorbide F‑Diagnose vorlag.

Die Datenanalyse erfolgte mithilfe des Statistikprogramms STATA (Version 16, StataCorp, College Station, Texas).

## Ergebnisse

### Stichprobenbeschreibung

Von den 1000 Anträgen enthielten 521 (52 %) eine F32- oder F33-Diagnose. Die soziodemographischen Merkmale der Patienten, von denen die Anträge stammen, können Tab. 1 entnommen werden. Die Zahl der F‑Diagnosen insgesamt reichte von 1 (55 % aller Anträge) bis 5 (0,6 %). Insgesamt 45 % hatten mehr als eine F‑Diagnose, 13 % hatten mehr als zwei F‑Diagnosen.

**Table 1:** 

		*N*	**%**
Geschlecht	Männlich	116	22
	Weiblich	405	78
Alter	< 30 Jahre	84	16
	30–39	141	27
	40–49	141	27
	50–59	125	24
	60–69	25	5
	70+	5	1
Bildung	Kein Schulabschluss	5	1
	Hauptschule	57	11
	Realschule/mittlere Reife	114	22
	Polytechnische Oberschule	7	1
	Fachhochschulreife	27	5
	Abitur	186	36
	Anderer Schulabschluss	2	0,4
	Unbekannt/nicht eindeutig	123	24
Krankenversicherung	Gesetzlich	501	96
	Privat	1	0,2
	Beihilfe	18	3
	Sonstiges (z. B. Heilfürsorge)	1	0,2

Von den 521 eingeschlossenen Anträgen waren 20 Anträge auf Kurzzeittherapie, 128 Erstanträge auf Langzeittherapie, 205 Umwandlungsanträge und 168 Fortführungsanträge (siehe Tab. 2). Gruppentherapie wurde in 26 der Fälle beantragt, bei allen anderen Fällen handelte es sich um Einzeltherapie. Aus diesem Grund wurde bei den Zusammenhangsanalysen von Schweregrad und Kontingent nicht hinsichtlich Einzel- vs. Gruppentherapie stratifiziert. Es gab keine Hinweise darauf, dass der Schweregrad mit der Beantragung von Gruppen- versus Einzeltherapie zusammenhing (*p* = 0,37).

**Table 2:** 

		*N*	**%**
Antragsart	KZT Erstantrag	19	4
	KZT erneuter Antrag	1	0,2
	LZT Erstantrag	128	25
	LZT Umwandlungsantrag	205	39
	LZT Fortführungsantrag	168	32
Beantragtes Verfahren	Tiefenpsychologisch fundierte Psychotherapie	410	79
	Analytische Psychotherapie	111	21
Abrechnungsgenehmigung des Therapeuten	Tiefenpsychologisch fundierte Psychotherapie	379	73
	Analytische Psychotherapie	37	7
	Analytische Psychotherapie und tiefenpsychologisch fundierte Psychotherapie	91	17
	Verhaltenstherapie und tiefenpsychologisch fundierte Psychotherapie	7	1
	Alle drei Verfahren	7	1

*KZT* Kurzzeittherapie*, LZT* Langzeittherapie

Die Diagnosen waren 63× als leicht kodiert (12 %), 349× als mittelgradig (67 %), 50× als schwer (10 %), 4× als schwer mit psychotischen Symptomen (1 %), 1× als remittiert, 2× als sonstige und 52× als „nicht näher bezeichnet“. Aufgrund der geringen Zahl von Patienten mit psychotischen Symptomen wurden in den Korrelationsanalysen die beiden Gruppen von schwer Depressiven zusammengefasst.

Von den leicht Depressiven hatten 52 % eine weitere F‑Diagnose, von den mittelgradig Erkrankten 43 % und von den schwer Erkrankten 48 %.

Es gab keine Hinweise auf Unterschiede zwischen den beantragten Therapieverfahren (tiefenpsychologisch fundiert versus analytisch) hinsichtlich der Häufigkeit der Schweregrade (*p* = 0,33).

### Zahl der beantragten und der befürworteten Stunden nach Schweregrad

Durchschnittlich wurden 80 h *beantragt* (Median 50, Spannweite 10–327, Standardabweichung (SD) 52) und 76 h *befürwortet* (Median 50, Spannweite 0–327, SD 52). In insgesamt 14 Fällen (3 %) wurde der Antrag nicht befürwortet, in 37 weiteren Fällen wurden weniger Stunden befürwortet als beantragt.

**Figure 1:**
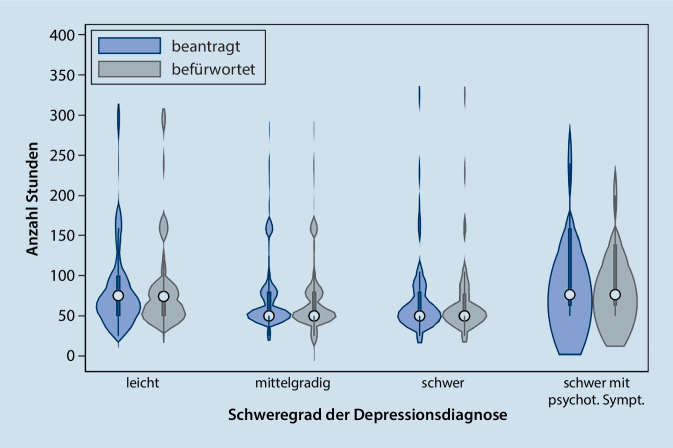

Bei leicht Erkrankten wurden im Median 75 h *beantragt *(bei einer Spannweite von 25 bis 300 h), bei mittelgradig Erkrankten 50 h (Spannweite 25 bis 286 h), bei schwer Erkrankten ebenfalls 50 h (25 bis 327 h) und bei Erkrankten mit psychotischen Symptomen 76 h (50 bis 240). Die Korrelation zwischen Schweregrad und beantragten Stundenkontingenten lag bei −0,10 (Abb. 1). Es gab keine Hinweise auf statistisch überzufällige Unterschiede im Median (Pearsons χ^2^ = 1,9, *p* = 0,37). Bei den Anträgen auf tiefenpsychologisch fundierte Psychotherapie lag der Korrelationskoeffizient bei −0,08, bei denen auf analytische Psychotherapie bei 0,01.

Die Zahl der *befürworteten* Stunden lag bei leichten Depressionen im Median bei 74 (Range 25 bis 300 h), bei mittelgradigen Depressionen bei 50 (0 bis 286 h), bei schweren Depressionen bei 50 (25 bis 327 h) und bei schweren Depressionen mit psychotischer Symptomatik bei 76 (50 bis 200). Die Korrelation betrug −0,11; es gab geringe Hinweise auf Unterschiede im Median (Pearsons χ^2^ = 6,5, *p* = 0,04). Bei den Anträgen auf tiefenpsychologisch fundierte Psychotherapie lag der Korrelationskoeffizient bei −0,08, bei denen auf analytische Psychotherapie bei −0,02.

### Sensitivitätsanalyse: beantragte und befürwortete Stunden nur bei Erstanträgen

Betrachtet man lediglich die Erstanträge (*n* = 148), findet man im Median 50 beantragte und ebenso auch 50 befürwortete Stunden in jeder Schweregradgruppe. Der Zusammenhang von Schweregrad mit Stundenzahl war erneut gering, sowohl bei den beantragten (Rho = −0,14; χ^2^ = 1,9 mit *p* = 0,40) als auch bei den befürworteten Stunden (Rho = −0,17, χ^2^ = 6,7 mit *p* = 0,04).

### Sensitivitätsanalyse: beantragte und befürwortete Stunden nur bei Patienten ohne komorbide psychische Erkrankungen

Wenn man nur jene Patienten analysiert, die außer der Depressionsdiagnose keine weitere F‑Diagnose hatten (*n* = 287), wurden bei leichter Depression im Median 80, bei mittelgradiger Depression 50 und bei schwerer Depression ebenso 50 h beantragt und auch befürwortet. Der Korrelationskoeffizient für den Zusammenhang von Schweregrad mit Stundenzahl lag bei −0,08 (*p* = 0,16) für beantragte sowie bei −0,09 (*p* = 0,13) für befürwortete Stunden.

## Diskussion

Diese Studie ging der Frage nach, ob das Kontingent an beantragten bzw. vom Gutachter befürworteten Stunden damit korreliert, als wie schwer die depressive Erkrankung von den Psychotherapeuten kodiert wird. Es gab in den Anträgen auf Kostenübernahme von Psychotherapie keine Hinweise auf einen solchen linearen Zusammenhang, der einer Zuweisung von Kontingenten allein aufgrund des Schweregrads der Diagnose entsprechen würde. Im Gegenteil, es zeigt sich auch innerhalb der Diagnosegruppen eine große Spannweite an Stundenkontingenten. Wie ist das zu erklären?

Zunächst einmal muss gefragt werden, ob die Fallzahl überhaupt in der Lage war, relevante Unterschiede zu entdecken, also gegen den statistischen Zufall abzusichern. Wir hatten *a priori* entschieden, dass ein Unterschied von 25 h relevant sein würde. Mit der vorliegenden Fallzahl (63 leicht, 349 mittelgradig und 54 schwer Erkrankte), der gegebenen Varianz und einem Alpha von 0,01 kann dieser Unterschied mit einer Power von 0,95 gegen den Zufall abgesichert werden. Dies bedeutet, dass die Fallzahl groß genug war, um den als relevant erachteten Unterschied statistisch entdecken zu können.

Eine weitere Einschränkung ist, wie oben erwähnt, dass bei der Stichprobe zwar die bereits stattgefundenen und die im aktuellen Bewilligungsschritt beantragten Stunden berücksichtigt werden konnten, nicht jedoch a) die später noch folgenden und b) die bei anderen Therapeuten im Vorfeld durchgeführten Kontingente. Das Maximum der Stunden dürfte also höher liegen. Dies würde dann die Ergebnisse beeinflusst haben, wenn bei Patienten mit Depressionen unterschiedlichen Schweregrades die Höhe der (nicht analysierbaren) später noch folgenden bzw. Vorbehandlungskontingente unterschiedlich wäre. Dies kann weder belegt noch ausgeschlossen werden. Abrechnungsdaten von Krankenkassen wären ein alternativer Zugangsweg zu Informationen über Stundenkontingente. Das hätte den Vorteil, dass die Stunden therapeutenübergreifend pro Patient kumuliert werden könnten. Eine Schwierigkeit besteht hier allerdings darin, kassenübergreifend Daten zusammenzuführen. Wenn man sich aber nur auf einzelne Kassen bezieht, besteht wiederrum die Gefahr einer Selektion von bestimmten Patientengruppen. Darüber hinaus sind bei den meisten Krankenkassen vermutlich nur die abgerechneten Stundenkontingente gespeichert, nicht aber die beantragten, welche wiederum in den Psychotherapieanträgen enthalten sind.

Einschränkend muss außerdem bedacht werden, dass Kurzzeittherapien, die nicht in eine Langzeittherapie umgewandelt wurden und bei denen der Therapeut von der Gutachtenpflicht befreit war, in dem Pool unserer Anträge fehlen. Dies würde dann die Ergebnisse beeinflusst haben, wenn diese Tatsache mit dem Schweregrad der Diagnose zusammenhängt. Auch dies kann weder ausgeschlossen noch belegt werden.

Abgesehen davon könnte ein Problem dann vorliegen, wenn die in der Studie eingeschlossenen Patienten nicht repräsentativ für die Gesamtpopulation von depressiv Erkrankten in Psychotherapie sind, denn eine Einschränkung unserer Studie besteht darin, dass wir keine Anträge auf Verhaltenstherapie zur Verfügung hatten. Dies ist aber nicht sehr wahrscheinlich, da die Verteilung von Alter, Geschlecht und Bildung in unserer Stichprobe derjenigen einer repräsentativen Studie von Psychotherapiepatienten [1] sowie derjenigen einer vergleichbaren Analyse von Psychotherapieanträgen [13] entspricht. Es ist jedoch möglich, dass das Spektrum und Muster von Komorbiditäten bei Patienten in Verhaltenstherapie versus in psychodynamischen Verfahren unterschiedlich ist. Es ist schwierig, dies genau zu überprüfen, da die Vielzahl möglicher Kombination von Diagnosen sehr hoch ist, besonders, wenn man bedenkt, dass häufig auch mehr als zwei F‑Diagnosen vergeben werden (in unserer Studie bei 13 % aller Fälle, im der Studie zur Gesundheit Erwachsener in Deutschland sogar bei 23 % aller Fälle [10]) und dass zusätzlich körperliche Erkrankungen unterschiedlichen Schweregrades vorliegen können. Allein diese Komplexität legt jedoch schon nahe, dass eine Stundenkontingentierung allein anhand einer einfachen F‑Diagnose zumindest zu problematisieren ist. Dass Komorbiditäten häufig und für die Behandlung relevant sind, ist gut belegt [3, 6, 15, 19, 22]. Prinzipiell stellt sich die Frage, wie der Schweregrad einer psychischen Erkrankung festgestellt werden kann. Für die in unserer Studie verwendeten F32- und F33-Diagnosen muss dies vom Psychotherapeuten festgelegt werden, für die meisten anderen F‑Diagnosen hingegen nicht. Wenn man ein transdiagnostisches Verfahren verwenden könnte, würde man das Problem der multiplen Komorbiditätsmuster umgehen. Ansätze dafür bieten Überlegungen zur dimensionalen Erfassung von Persönlichkeitsfunktionen [23, 25], das Modell der Hierarchical Taxonomy of Psychopathology [8, 17] sowie Strukturkonzepte [16, 24]. Hier sind noch weitere konzeptionelle und empirische Arbeiten notwendig.

Der Anteil von analytischen bzw. tiefenpsychologisch fundierten Therapien in unserer Studie ähnelt dem in publizierten Daten [13]. Deshalb gehen wir davon aus, dass unsere Ergebnisse eine klinische Wirklichkeit widerspiegeln, wie sie in der Praxis tatsächlich gegeben ist. Demnach orientieren sich die Psychotherapeuten (und auch die Gutachter) bei der Einschätzung dessen, wie viele Stunden für eine Psychotherapie notwendig sind, nicht nach groben Kriterien der F‑Diagnose, sondern nach anderen, vermutlich komplex interagierenden Gesichtspunkten (z. B. Komorbiditätsmuster, Strukturniveau, Änderungsmotivation usw. [12]). Unsere Daten zeigen, dass die Varianz bei den beantragten und befürworteten Kontingenten innerhalb einer Diagnosekategorie hoch ist, was eine solche Interpretation nahelegt. Demnach prüfen die Psychotherapeuten in Zusammenarbeit mit den Patienten im Einzelfall, wie viele Stunden an Therapie zum gegenwärtigen Zeitpunkt notwendig sind. In eine ähnliche Richtung weisen Befunde der MARS-Studie, in der sich zeigte, dass die Morbidität und die Symptomatik von Psychotherapiepatienten nicht damit in Zusammenhang stand, ob ein Erst- oder Umwandlungsantrag gestellt wurde [13].

Auch eine Studie basierend auf dem Mental Health Survey der Weltgesundheitsorganisation kam zu dem Schluss, dass eine alleinige Berücksichtigung des Schweregrads einer Erkrankung zu kurz greift, um die Krankheitslast beurteilen zu können [2].

Grundsätzlich besteht eine Limitation unserer Studie natürlich darin, dass wir die gegenwärtige Versorgungspraxis analysiert haben. Daraus kann man nicht notwendigerweise ableiten, dass dies die beste Art und Weise ist, die begrenzten Ressourcen optimal zu verteilen. Man kann zwar wohl davon ausgehen, dass Psychotherapeuten und Patienten auch jetzt schon gemeinsam abwägen, wie viele Stunden sie für die Therapie benötigen, aber das bedeutet nicht zwangsläufig, dass es dann immer auch so beantragt wird. So ist beispielsweise gut denkbar, dass Patienten eigentlich noch weitere Stunden benötigen, aber die Therapeuten dies nicht empfehlen, da die Kontingentgrenzen erreicht sind.

## Fazit für die Praxis

Die Ergebnisse sprechen aus unserer Sicht gegen eine Politik der Zuordnung von Stundenkontingenten für Psychotherapien allein anhand der Diagnose, zumindest entspricht dies nicht der gegenwärtigen Versorgungspraxis. Es muss also auf andere Weise erarbeitet werden, wie die vorhandenen Mittel der Solidargemeinschaft am zweckmäßigsten genutzt werden können, damit alle Menschen, die eine fachpsychotherapeutische Behandlung benötigen, diese auch in ausreichendem Maße erhalten. Dies sollte wissenschaftlich fundiert und Empirie-basiert erfolgen.
